# Prevalence of chronic kidney disease and associated risk factors among people living with HIV in a rural population of Limpopo Province, South Africa

**DOI:** 10.3389/fpubh.2024.1425460

**Published:** 2024-07-11

**Authors:** Joel Choshi, Brian Flepisi, Sihle E. Mabhida, Machoene D. Sekgala, Haskly Mokoena, Bongani B. Nkambule, Duduzile Ndwandwe, Zandile J. Mchiza, Unati Nqebelele, André P. Kengne, Phiwayinkosi V. Dludla, Sidney Hanser

**Affiliations:** ^1^Department of Physiology and Environmental Health, University of Limpopo, Sovenga, South Africa; ^2^Department of Pharmacology, University of Pretoria, Pretoria, South Africa; ^3^Non-Communicable Diseases Research Unit, South African Medical Research Council, Tygerberg, South Africa; ^4^School of Laboratory Medicine and Medical Sciences, University of KwaZulu-Natal, Durban, South Africa; ^5^Cochrane South Africa, South African Medical Research Council, Tygerberg, South Africa; ^6^School of Public Health, University of the Western Cape, Bellville, South Africa; ^7^Department of Medicine, University of Cape Town, Cape Town, South Africa; ^8^Department of Internal Medicine, University of the Witwatersrand, Johannesburg, South Africa; ^9^Department of Biochemistry and Microbiology, University of Zululand, KwaDlangezwa, South Africa

**Keywords:** chronic kidney disease, people living with HIV, individuals without HIV, prevalence, risk factors

## Abstract

**Background:**

Limited evidence informs on the prevalence of chronic kidney disease (CKD) in people living with HIV (PLWH) in South Africa. Thus, this study aimed to determine the prevalence of CKD and its associated risk factors among PLWH within the rural province of Limpopo, South Africa.

**Methods:**

We conducted a cross-sectional study of 143 participants, subdivided into groups of PLWH (*n* = 103) and individuals without HIV (*n* = 43). Structured questionnaires were used to collect and capture sociodemographic information including age, sex, alcohol intake, smoking status, and educational status. Basic measurements taken included levels of cluster of differentiation 4 (CD4+) count, body mass index (BMI), blood pressure, plasma cystatin C, and fasting serum glucose levels. Plasma cystatin C-based estimated glomerular filtration rate (eGFR) was calculated using the chronic kidney disease epidemiology collaboration (CKD-EPI) estimator to determine the prevalence of CKD.

**Results:**

The prevalence of CKD was approximately 7% in PLWH. Multivariate logistic regression analysis showed that it was only diabetes mellitus (odds ratio of 5.795, 95% confidence interval, *p* = 0.034) and age (odds ratio of 1.078, 95% confidence interval, *p* = 0.039) that were significantly associated with CKD in PLWH.

**Conclusion:**

Chronic kidney disease was prevalent in PLWH, and it was further associated with cardiovascular risk factors, diabetes, and ageing. As PLWH age, the burden of CKD may be increased with the increase in cardiovascular-related comorbidities such as diabetes.

## 1 Introduction

Chronic kidney disease (CKD) is one of the highly prevalent non-infectious comorbidities that significantly contributes to mortality among people living with HIV (PLWH) (1). People living with HIV continue to experience a rise in the prevalence of CKD despite antiretroviral therapy, and this presents a significant challenge to healthcare systems in both developed and developing countries (2). The risk of developing CKD is higher in PLWH than in the general population, with the condition likely to progress to end-stage renal disease (ESRD) at a 2- to 20-fold higher risk (2, 3). The heightened risk is mainly due to HIV-induced renal injury, diminished CD4+ counts, treatment-related effects, and the presence of comorbidities such as diabetes and hypertension (4). In the ageing PLWH with CKD, there is an increasing co-existence of comorbidities, predominantly including diabetes, hypertension, and obesity (5) which contribute to CKD progression and poor health outcomes. The prevalence of CKD in PLWH tends to vary by geographical location, the type of estimating method, diagnostic criteria, and population characteristics (6).

One systematic review and meta-analysis reported overall CKD prevalence of 6.4, 4.8, and 12.3% among PLWH by modification in the diet in renal disease (MDRD), chronic kidney disease–epidemiology collaboration (CKD-EPI), and Cockcroft–Gault equations, respectively (7). Africa showed the highest CKD prevalence at 7.9% in PLWH according to the MDRD formula, and within the African continent, CKD prevalence was highest in West Africa at 14.6% and lowest in Southern Africa at 3.2% according to the MDRD formula (7). Observational studies within Sub-Saharan Africa also reported varying CKD prevalence ranging from 2.5 to 44%, where the lowest CKD prevalence and the highest CKD prevalence were found in Uganda and Cameroon (8, 9). Diabetes and hypertension are known to be the major driving forces for the development and progression of CKD in both the general population and PLWH (10). The higher prevalence of CKD in PLWH in Africa may also be attributed to the genetic susceptibility of people of African descent, who have an 18- to 50-fold higher risk of developing HIV-associated ESRD compared to Caucasians (11–13). South Africa has one of the highest prevalence rates of HIV globally (14), and studies reporting on the prevalence of CKD in PLWH in South Africa are scanty, underscoring the need for studies to determine the prevalence of CKD in this high-risk population. As the prevalence of CKD is often associated with hypertension, diabetes, age, obesity, and low CD4+ counts in PLWH (10, 15, 16), it is also essential to determine whether these risk factors are associated with the presence of CKD among PLWH in South Africa.

## 2 Methods

### 2.1 Study design and ethical approval

This cross-sectional study was conducted in the Mankweng district of Limpopo Province, South Africa. The participants were recruited from 02 October 2019 to 02 October 2020. The study was approved by the Turfloop Research and Ethics Committee (TREC) (project number TREC/315/2019: PG), as part of the initial project approved by TREC (project number TREC/119/2016: PG). The study adhered to the principles of the Declaration of Helsinki (17), and informed consent was obtained from participants.

### 2.2 Study population, sampling technique, and eligibility criteria

**Figure 1:**
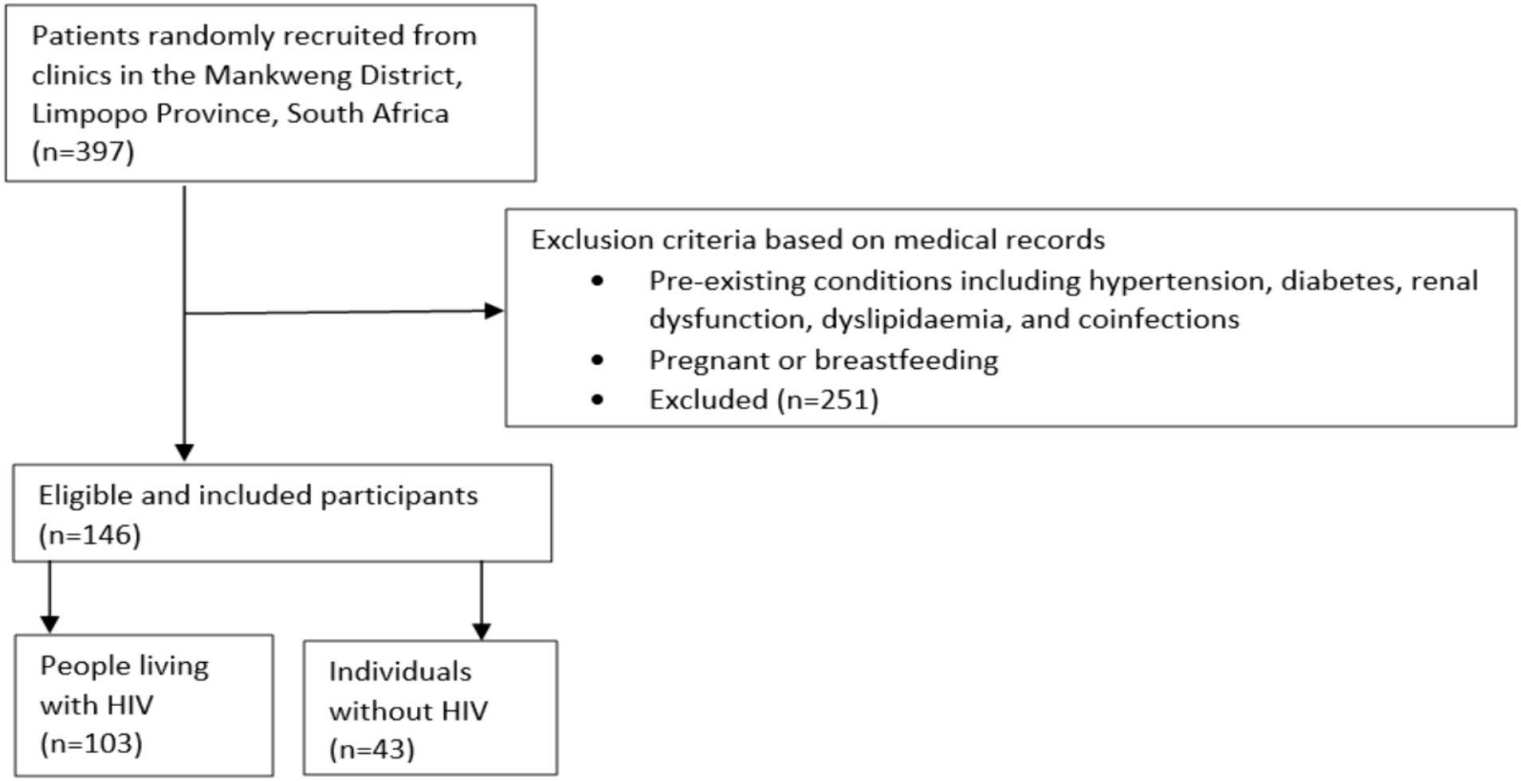
Flow diagram presents participant selection. Briefly, 397 patients were identified at local clinics in Mankweng District, Limpopo Province, South Africa. A total of 146 participants were included after excluding those with comorbidities and subdivided into 103 people living with HIV and 43 individuals without HIV.

A total of 146 participants (≥18 years) including men and women were enrolled in the study (Figure 1). The overall population (*n* = 146) consisted of PLWH (*n* = 103) and individuals without HIV (*n* = 43). The sample size was determined using a formula developed by Cochran (18), which considers a 5% margin error and 95% confidence level. Men and women aged ≥18 years who signed the informed consent form were allowed to participate. However, individuals found to be presenting with other comorbidities including renal dysfunction, hypertension, diabetes, dyslipidaemia, and co-infections based on medical records at the time of enrolment, as well as those who were pregnant or breastfeeding, were excluded.

### 2.3 Demographic data and medical information

Upon obtaining informed consent, structured questionnaires were used to collect data on sociodemographic and clinical characteristics as well as pre-existing medical conditions through screening of medical records. Anthropometric measurements including weight and height were measured using standardised protocols, as previously described (19). Body mass index (BMI) was calculated as weight in kilograms (kg) divided by the square of the height in metres (m). The BMI was further classified as underweight, normal, overweight, and obesity using the Centers for Disease Control and Prevention cutoffs (20). Blood pressure was measured using a digital Omron M2 monitor (OMRON Healthcare, Japan) according to the manufacturer’s instructions. The Southern African Hypertension Guidelines were used to classify blood pressure (21).

### 2.4 Blood collection and biochemical analysis

A qualified nurse collected blood once-off from participants. Fasting venous blood was drawn from the median cubital vein and then transferred into ethylenediaminetetraacetic acid (EDTA) and BD vacutainer tubes (Becton Dickinson, NJ, United States). Whole blood samples were used to determine the CD4+ count at a laboratory using a factory-calibrated Alere PIMA analyser (Alere Technologies GmbH, Germany) as per the manufacturer’s instructions. The CD4+ count was classified into stages using the reference ranges described by Garcia and Guzman (22). The blood samples were further processed into serum and plasma samples using an Allegra X-30 benchtop centrifuge (Beckman Coulter, IN, United States) at 3,000 RMP and 18°C for 20 min. Serum glucose was analysed using Cobas Integra^®^ 400 plus analyser (Roche Holding AG, Basel, Switzerland) according to the manufacturer’s instructions. Diabetes was confirmed with the American Diabetes Association (ADA) criteria (23). Cystatin C was analysed in plasma samples using the Milliplex^®^ map human kidney injury magnetic bead panel 6 assay on Luminex^®^ xMAP^®^ instrument (Merck KGaA, Darmstadt, Germany), according to the manufacturer’s instructions. Plasma cystatin C concentrations were used to determine eGFR using the CKD-EPI formula to determine the presence of CKD, according to the Kidney Disease: Improving Global Outcomes (KDIGO) guidelines (24). Chronic kidney disease was defined as an eGFR < 60 mL/min/1.73 m^2^ (25).

### 2.5 Statistical analysis

Statistical analysis was conducted on the Statistical Package for the Social Sciences (SPSS) software version 29 (IBM Corporation, USA). The normality test was performed to check the distribution of the data. The included continuous variables (age and CD4+ count) showed a Gaussian distribution. Descriptive statistics were performed, and continuous data were presented as means and standard deviations, while categorical variables were presented as frequencies and percentages. The independent-samples *t*-test was performed to compare the means across study groups for continuous variables while the chi-square test was used to compare the percentages for categorical variables. A logistic regression analysis was also performed to determine the factors associated with CKD. The significance difference and association level were assumed at *p*-value <0.05.

## 3 Results

### 3.1 Demographics and clinical characteristics of the study population

The present study enrolled a total population of 146 black African adult participants consisting of PLWH (*n* = 43) and individuals without this condition (*n* = 103). The sociodemographic and clinical characteristics of the study population are presented in Table 1. The presented data showed that PLWH had a significantly higher mean age (41.91 ± 10.84) than individuals without HIV (37.09 ± 16.59) (*p* = 0.040). Individuals without HIV had a higher proportion of women (67.4%) than men (32.6%). Similarly, PLWH had a higher proportion of women (65%) than men (25%). The CD4+ count was 407.00 ± 240.87 in PLWH, whereas approximately 22% of these individuals were in the advanced HIV stage as indicated by CD4+ count <200 cells/μL. A significantly higher proportion of PLWH consumed alcohol (27.2%) as compared to individuals without HIV (9.3%). Obesity, diabetes, and hypertension were not significantly different between the two study groups. The levels of smoking and educational status were also not significantly different between the two study groups.

**Table 1:** Sociodemographic and clinical characteristics of the study population.

	Individuals without HIV	PLWH	*p*
	*n* = 43	*n* = 103	
Age (years)	37.09 ± 16.59	41.91 ± 10.84	**0.040**
Age group, *n* (%)			
18–29	18 (41.9)	14 (13.6)	
30–39	13 (30.2)	34 (33.0)	
40–49	3 (7.0)	31 (30.1)	**<0.001**
≥50	9 (20.9)	24 (23.3)	
Sex, *n* (%)			
Female	29 (67.4)	67 (65.0)	0.781
Male	14 (32.6)	36 (35.0)	
CD4+ count (cells/μL)	–	407.00 ± 240.87	**–**
CD4+ category, *n* (%)			
≥200	–	72 (78.3)	**–**
<200	–	20 (21.7)	
Marital status, *n* (%)			
Single	27 (62.8)	53 (51.5)	
Married	10 (23.3)	26 (25.2)	0.359
Cohabiting	6 (14.0)	24 (23.3)	
Education status, *n* (%)			
No formal education	1 (2.3)	7 (6.8)	
Primary	6 (14.0)	14 (13.6)	0.590
Secondary	30 (69.8)	73 (70.9)	
Tertiary	6 (14.0)	9 (8.7)	
Alcohol consumption, *n* (%)	4 (9.3)	28 (27.2)	**0.017**
Smoking, *n* (%)	7 (16.3)	25 (24.3)	0.287
BMI category (kg/m^2^), *n* (%)			
Underweight	0	8 (7.8)	0.052
Normal	17 (39.5)	40 (38.8)	
Overweight	9 (20.9)	32 (31.1)	
Obese	17 (39.5)	23 (22.3)	
Diabetes, *n* (%)	5 (11.6)	14 (13.6)	0.748
Hypertension, *n* (%)	7 (16.3)	25 (24.3)	0.287

HIV, human immunodeficiency virus; CD4+, cluster of differentiation 4. Continuous data are expressed as mean ± standard deviation and categorical data as frequency and percentage. The bold text indicates *p*-values <0.05.

### 3.2 Prevalence of chronic kidney disease in PLWH

**Figure 2:**
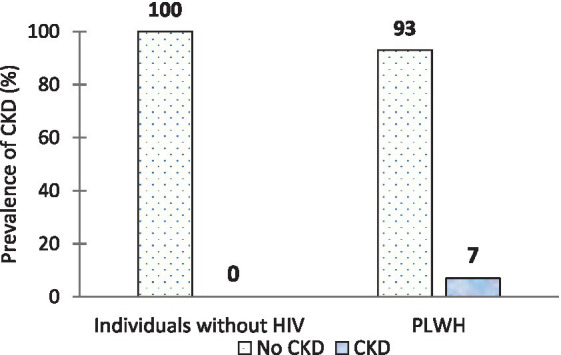
Prevalence of chronic kidney disease in people living with HIV in comparison with individuals without this condition.

Cystatin C-based eGFR was used to determine the prevalence of CKD in both PLWH and individuals without HIV in the present study. The prevalence of CKD was found to be 7% in PLWH, while no participant reported CKD in the individuals without HIV group (Figure 2).

### 3.3 Factors associated with chronic kidney disease in PLWH

The logistic regression analysis was performed to find any association between CKD and diabetes, age, hypertension, BMI ≥ 25 kg/m^2^, alcohol use, and advanced HIV stage (CD4+ count <200). The results showed that diabetes (crude odd ratio = 5.795, confidence interval: 1.143–29.38; *p* = 0.034) and age (crude odd ratio = 1.078, confidence interval: 1.004–1.158, *p* = 0.039) were significantly associated with CKD in the crude analysis (Table 2). Both diabetes and age as well as hypertension, BMI ≥ 25 kg/m^2^, alcohol use, and advanced HIV stage did not show any association with CKD after adjusting for sex, tobacco use, educational status, and marital status.

**Table 2:** Logistic analysis of risk factors associated with chronic kidney disease in PLWH.

		COR (95%CI)	*p*	AOR (95%CI)	*p*
Diabetes	No (ref)	5.795 (1.143–29.38)	**0.034**	6.340 (0.901–44.597)	0.064
	Yes				
Age		1.078 (1.004–1.158)	**0.039**	1.109 (0.984–1.184)	0.107
Hypertension	No (ref)	1.720 (0.231–6.988)	0.784	1.604 (0.258–9.996)	0.613
	Yes				
BMI	<25 (ref)	0.635 (0.135–2.990)	0.565	1.029 (0.153–6.903)	0.977
	≥25				
Alcohol consumption	No (ref)	2.130 (0.445–10.185)	0.344	2.559 (0.400–16.369)	0.321
	Yes				
CD4+ count	≥200 (ref)	0.705 (0.078–10.185)	0.344	2.559 (0.400–16.369)	0.321
	<200				

Variables adjusted: sex, tobacco use, educational status, and marital status. COR, crude odds ratio; CD4+, cluster of differentiation 4; AOR, adjusted odd ratio; Cl, confidence interval; *p*, significance level. The bold values indicate *p*-values < 0.05.

## 4 Discussion

The results for demographic characteristic features of participants showed that the mean age for PLWH was significantly higher than those without this condition. This finding currently has no statistical value as individuals without HIV were mostly young adults. Most of the PLWH in the present study were in the age group of 30–39 years, which supports the studies by Boshomane et al. (26) and Johnson and Dorrington (27) which showed that HIV is mostly prevalent among those aged ≥25 years, 30–34 years, or ≥35 years in the Limpopo Province. There were more women living with HIV than men, consistent with recent findings by Johnson and Dorrington (27), which showed a disproportionately higher prevalence of HIV in women than in men in Limpopo Province. This may be attributed to the heightened risk in women due to socioeconomic, behavioural, and biological factors (28). For instance, women are mostly likely to find themselves unemployed and in poverty, which promotes unsafe sex practises, such as engaging in transactional sex with casual partners without protection (29). Our results also showed a higher proportion of PLWH who are single, corroborating previous findings that report an increased risk of HIV among single individuals compared to married or cohabiting individuals due to their likelihood of engaging in risky behaviours such as having multiple partners and engaging in transactional sex (26, 30, 31). In terms of educational status, this study showed that most of the PLWH were at the secondary school level. On the contrary, a previous study reported a higher HIV prevalence among people with primary education or no formal education than those with secondary education in the Limpopo Province (26). Despite this, our finding reflects higher HIV transmission rates in the early adolescent stages when they were still in school, possibly due to sexual experimentation and engaging in unsafe sex practises with multiple partners.

We found a higher prevalence of CKD in PLWH than in HIV-uninfected individuals (no CKD case), which is comparable with the overall CKD prevalence of 7.9% in PLWH in Africa (7). Our finding is also comparable with the findings of Gunter et al. (32) from Belgium and Goulet et al. (33) from the United States who reported CKD prevalence rates of 7.8 and 7% in HIV-infected individuals using creatinine-based measures. In the logistic analysis, diabetes and age were associated with CKD in our PLWH, suggesting that as the HIV population ages, comorbidities such as diabetes develop and become prevalent, predisposing individuals to the risk of developing CKD. A similar study by Debeb et al. (34) in Ethiopia also showed that older age and diabetes were significantly associated with CKD in PLWH. Our CKD prevalence in PLWH was lower than the prevalence reported by Calza et al. (35) from Italy (21.3%), which was associated with multiple risk factors including older age, hypertension, diabetes, male gender, low nadir CD4+ count, hypertriglyceridemia, and use of tenofovir. In addition, Calza et al. (35) used a sensitive CKD diagnostic criterion incorporating urinalysis, which could explain this disparity. The majority of our PLWH were not in the advanced HIV stage and were under the 50-year age group, which could also explain the disparity with Calza et al. (35) study. On the other hand, we found a much higher CKD prevalence than Nyende et al. (8) from Uganda (2.5%), who attributed the low prevalence to the small sample size and not receiving critically ill outpatients.

Ageing and diabetes may explain the higher prevalence of CKD in our PLWH. Diabetes is a well-recognised traditional risk factor for CKD (10) and contributes to renal disease via hyperfiltration stress, hyper-reabsorption stress, or endothelial dysfunction (36). On the other hand, ageing is a strong unmodifiable risk factor for CKD among PLWH and as they age, comorbidities increase, and CKD becomes more prevalent (2). Ageing is also known to facilitate renal function deterioration in both the general population and PLWH, which is related to increased levels of oxidative stress resulting in structural and functional changes in the kidney (37). The higher prevalence of CKD in our PLWH may also be explained by the genetic predisposition that HIV-infected Africans have for HIV-related renal disease. People of African descent are known to carry APOL1 kidney disease risk variants, which are strongly associated with the progression of HIV-associated nephropathy to CKD and ESRD during HIV infection (38). In addition to the traditional risk factors such as ageing and diabetes, PLWH is also susceptible to other comorbidities, especially Hepatitis C infections, which may also account for the higher prevalence of CKD in PLWH, as explained by Ranivoharisoa et al. (39). Moreover, HIV infection has a direct effect on causing renal function decline, and the detrimental effect of antiretroviral medication further contributes to an increased risk of developing CKD among the PLWH (4). Our finding suggests that PLWH may soon experience ESRD and warrants further investigation.

Other risk factors including hypertension, BMI ≥ 25 kg/m^2^, alcohol use, and advanced HIV stage were not significantly associated with CKD in our population, which may be explained by the fact that few had CD4+ count <200 cells/μL and were in ≥50-year age group. The risk factors correlate well with CKD among older patients (40). The study is limited by its cross-sectional nature which makes it impossible to infer causation. The disparity in the number of individuals between the study groups may have influenced the outcomes of the study. The current study observed a significant association between age and diabetes with CKD in the crude analysis, which did not persist after adjusting for some potential confounders. This may reflect that these risk factors were masked by other consequences, requiring further elaboration in future studies. Therefore, the high presence of CKD among the PLWH may reflect the interaction of risk factors, rather than their independent contributions in increasing the risk of CKD among PLWH. Furthermore, this study was conducted in a single district in the Limpopo Province, which potentially limits the generalisability of the findings. Cross-sectional studies usually do not have a control group. This study did not compare the clinical data of PLWH with CKD and non-CKD. Although the study is not without limitations, the preliminary information proves vital in understanding risk factors associated with CKD among PLWH. Importantly, the results are vital to build on future research to understand the epidemiology of this condition in a Black African population, especially for countries in Southern Africa. Furthermore, the study encourages the potential use of cystatin C-based measures for determining the presence of kidney disease in this high-risk group of PLWH, as previously highlighted (41).

## 5 Conclusion

The prevalence of CKD is higher in PLWH than in individuals without HIV and is associated with ageing and diabetes. This suggests that the risk of future adverse renal outcomes may be increased in this HIV population. Future longitudinal studies incorporating larger sample sizes are needed to confirm these findings.

## Data availability statement

The original contributions presented in the study are included in the article/supplementary material, further inquiries can be directed to the corresponding authors.

## Ethics statement

The studies involving humans were approved by Turfloop Research Ethics Committee, University of Limpopo. The studies were conducted in accordance with the local legislation and institutional requirements. The participants provided their written informed consent to participate in this study.

## Author contributions

JC: Conceptualization, Data curation, Formal analysis, Investigation, Methodology, Writing – original draft, Writing – review & editing. BF: Supervision, Writing – review & editing. SM: Data curation, Formal analysis, Writing – review & editing. MS: Data curation, Formal analysis, Writing – review & editing. HM: Writing – review & editing. BN: Writing – review & editing. DN: Writing – review & editing. ZM: Writing – review & editing. UN: Writing – review & editing. AK: Writing – review & editing. PD: Conceptualization, Funding acquisition, Investigation, Methodology, Project administration, Resources, Supervision, Validation, Visualization, Writing – original draft, Writing – review & editing. SH: Conceptualization, Funding acquisition, Investigation, Methodology, Project administration, Resources, Supervision, Validation, Visualization, Writing – original draft, Writing – review & editing.
